# Serotonin Signaling Modulates Sexual Receptivity of Virgin Female *Drosophila*

**DOI:** 10.1007/s12264-022-00908-8

**Published:** 2022-07-05

**Authors:** Baoxu Ma, Rencong Wang, Yaohua Liu, Bowen Deng, Tao Wang, Fengming Wu, Chuan Zhou

**Affiliations:** 1 State Key Laboratory of Integrated Management of Pest Insects and Rodents, Institute of Zoology, Chinese Academy of Sciences, Beijing, 100101 China; 2 University of Chinese Academy of Sciences, Beijing, 100101 China; 3 Department of Plant Protection, Shanxi Agricultural University, Jinzhong, 30801 China; 4 Chinese Institute for Brain Research, Zhongguancun Life Sciences Park, Beijing, 102206 China; 5 Institute of Molecular Physiology, Shenzhen Bay Laboratory, Shenzhen, 518132 China

**Keywords:** Female sexual receptivity, Serotonin, 5-HT, *Fruitless*, Neurochemical, 5-HT receptors, *Drosophila*

## Abstract

The choice of females to accept or reject male courtship is a critical decision for animal reproduction. Serotonin (5-hydroxytryptamine; 5-HT) has been found to regulate sexual behavior in many species, but it is unclear how 5-HT and its receptors function to regulate different aspects of sexual behavior. Here we used *Drosophila melanogaster* as the model animal to investigate how 5-HT and its receptors modulate female sexual receptivity. We found that knockout of tryptophan hydroxylase (*Trh*), which is involved in the biosynthesis of 5-HT, severely reduced virgin female receptivity without affecting post-mating behaviors. We identified a subset of sexually dimorphic *Trh* neurons that co-expressed fruitless (*fru*), in which the activity was correlated with sexual receptivity in females. We also found that 5-HT_1A_ and 5-HT_7_ receptors regulate virgin female receptivity. Our findings demonstrate how 5-HT functions in sexually dimorphic neurons to promote virgin female receptivity through two of its receptors.

**Supplementary Information:**

The online version contains supplementary material available at 10.1007/s12264-022-00908-8.

## Introduction

Sexual behavior in *Drosophila melanogaster* is an excellent model in which to investigate the neuronal basis underlying social behavior because they are innate and robust [1–3]. Wild-type male and female flies can achieve copulation without social learning experiences during adulthood [4, 5]. *Drosophila* sexual behaviors include stereotypic male courtship rituals such as orienting to a female, extending an ipsilateral wing to produce courtship songs, tapping and licking the female, attempting copulation, and finally copulation [6, 7]. The neural circuit involved in male courtship behavior has been dissected in recent years owing to advances in genetic technology [8–11]. However, studies on female sexual behavior are far fewer than those on males.

Much progress has been made in recent years on how female flies perceive the presence of males and their courtship, and integrate auditory, olfactory, and mechanosensory cues, to decide whether to be receptive or not [12–15]. Such a decision is also dependent on the maturity and mating status of the female. Sexually immature females exhibit rejection behaviors by running away, flicking wings, or kicking the courting male [16, 17]. After sexual maturity, virgin females make the decision to copulate with courting males and exhibit sexual receptivity, which is a proxy metric to evaluate the willingness of females to mate [18–20]. Recently-mated females display post-mating behaviors by reducing receptivity and increasing egg-laying [8, 21, 22]. Female post-mating behaviors are triggered by the male seminal fluid peptide (sex-peptide, SP) and regulated by SP-responsive neurons which express fruitless (*fru*), doublesex (*dsx*), and pickpocket (*ppk*) [23–26]. Despite this progress in the sensory and integrative circuits for female sexual behavior [26–29], very little is known about how internal factors modulate virgin female receptivity.

Internal factors that modulate the function of neuronal circuits often use neurochemical systems including neuropeptides and neurotransmitters [30]. Serotonin (5-hydroxytryptamine; 5-HT), which is one of the highly-conserved neurotransmitters across species, is involved in a range of behaviors including cognition, reward, learning, and memory, as well as male and female sexual behavior [31–35]. Although 5-HT is known to be involved in mammal sexual behavior, its exact function in regulating sexual behavior is unclear. In *Drosophila*, 5-HT has also been shown to regulate a variety of complex behaviors including aggression, sleep, and feeding [36–38], but whether and how it regulates female sexual receptivity is unknown. Thus, it is of particular importance to investigate the function of 5-HT in female sexual receptivity using the *Drosophila* model.

Sexual behaviors in *Drosophila* are largely controlled by two pivotal regulatory genes, *fru* and *dsx*, that control most aspects of sexual development and behavior [10, 39, 40]. Sex-specific *dsx* transcripts are translated in both sexes to produce the sex-specific proteins Dsx^M^ or Dsx^F^, which control male and female differentiation, respectively [15, 41–43]. In contrast, *fru* proteins (Fru^M^) control male courtship and are male-specific [1, 5, 44, 45]. Although Fru^M^ proteins are not produced in females, neurons expressing the *fru* transcript (*fru*^*+*^) are crucial for female sexual receptivity, as silencing these *fru*^*+*^ neurons impairs female receptivity [44, 46]. Recent studies have also revealed the importance of *dsx*^*+*^ neurons in controlling virgin female receptivity and post-mating behaviors in mated females [24, 27, 47, 48].

In this study, we showed that 5-HT signaling modulates female sexual receptivity at both the molecular and the neural circuit levels. Knockout and knockdown of tryptophan hydroxylase (*Trh*), which is involved in the biosynthesis of 5-HT, decreased virgin female receptivity. Activation of the entire population of *Trh*^+^ neurons enhanced sexual receptivity in virgin females but had no effect on sexual receptivity in mated females. We identified a group of sexually dimorphic *Trh*^+^*fru*^*+*^ neurons in the posterior lateral protocerebrum (PLP) to be a crucial 5-HT-releasing site in the regulation of female sexual receptivity. Analysis of Ca^2+^ activity in 5-HT-PLP neurons revealed stronger activity in virgin flies than in mated flies. Furthermore, we found two 5-HT receptors, 5-HT_1A_ and 5-HT_7_, that might be crucial for female sexual receptivity.

## Materials and Methods

### Fly Culture and Strains

All *D. melanogaster* strains were reared on standard medium at 25°C and 60% humidity in a 12-h light/dark photoperiod unless otherwise described. All the knockout lines in this study for screening have been published [49]. The following strains were obtained from Dr. Yi Rao’s lab (Peking University, Beijing, China): *isoCS* (wild-type), *Trh-GAL4*, *elav-GAL4*;*UAS-dicer2*, *elav-GAL4*, and *UAS-5-HT1A*. The *UAS-stingerGFP* and *UAS-Redstinger* lines were gifts from Dr. Yufeng Pan’s lab (Southeast University, Nanjing, China). *UAS-PACα* was a gift from Dr. Yan Zhu’s lab (Institute of Biophysics, Chinese Academy of Sciences). The following strains were from the Bloomington *Drosophila* Stock Center: *UAS-Kir2.1* (BL#6596), TRIC (BL#61679), *UAS-mCD8-GFP* (BL#5137), *UAS-shi*^*ts*^ (BL#44222), and *UAS-Trh-RNAi* (BL#33612).

### Behavioral Assays

Female receptivity assays were conducted as previously described [15]. In brief, individual virgin females (8–10 days old) were paired with a naïve wild-type male courter (*isoCS*) (8–10 days old). Before they were paired, females and males were separately introduced into a two-layer courtship chamber (10 mm diameter × 3 mm height per layer), which was divided by a removable transparent strip. The assay was recorded with a resolution of 1280 pixels × 720 pixels (1.78:1) at 30 frames/s for 30 min using cameras (VIXIA HF R500, Canon, Tokyo, Japan). The number of receptive females and the time of receptivity for individual females were analyzed manually.

In the egg-laying assay, 3–4 virgin or mated females (~8 days old) were transferred to a vial with fresh medium left for 48 h at 25°C and 60% humidity under a 12-h light/dark cycle, and the number of eggs laid per female during 48 h was counted manually. To collect mated females, individual females were aspirated into the courtship chamber to allow copulation with a wild-type male before the egg-laying tests.

In the re-mating assay, we obtained mated females by pairing virgin females with wild-type males (both ~8 days old). The mated females were collected as above, transferred to food vials, and left for 48 h before re-mating tests with a new wild-type male of the same age for 1 h. The percentage of re-mating females was analyzed manually.

The locomotion assay was applied at 25°C and 60% humidity. Individual virgin females were transferred to the courtship chambers without males and recorded for 10 min. The locomotor speed was analyzed using MatLab software (MathWorks Inc., MA, USA) as described previously [50].

All behavioral assays were run from 11:00 to 15:00. The food medium was replaced every 2–3 days to ensure freshness.

### Light-induced Experiments

In *PACα* (photoactivated adenylyl cyclase α) experiments, flies were crossed on standard medium, and the vials were wrapped in aluminum foil to avoid light. Female progeny (<8 h) were isolated in darkness for 8–10 days. Prior to behavioral tests, *PACα-*expression was activated by blue light (420 nm, 1200 mW/cm^2^, 5 s; Denjoy, DY-400-4, Changsha, China).

In *CsChrimson* experiments, flies were crossed on 0.2 mmol/L retinal-containing medium (Sigma-Aldrich, St. Louis, USA) in darkness. Virgin females were immediately transferred to 0.4 mmol/L retinal-containing medium and isolated in darkness for 8–10 days. Female receptivity tests were performed in darkness (control) or with red light activation (620 nm, 0.03 mW/mm^2^; Kemai Vision Technology, Dongguan, China) during a 30-min observation period. The assay was recorded by an industrial camera (Stingray F080B ASG, Allied Vision Technologies, Stadtroda, Germany) equipped with an infrared light source (860-nm IR LED, Kemai Vision Technology) for illumination.

### Temperature-induction Experiments

In *TrpA1* and *UAS-shi*^*ts*^ experiments, virgin females were maintained at 22°C for 8–10 days. Before the behavioral assay, the flies were concurrently introduced into chambers at 30°C or 21°C for 20 min. 30°C was the activation temperature in *TrpA1* activation experiments but a restrictive temperature in *UAS-shi*^*ts*^ inactivation experiment.

### Generation of *UAS-5-HT7*

pJFRC28-5XUAS-IVS-GFP-p10 (# 12073; Fungene Biotechnology, Shanghai, China) was used for the generation of the pJFRC28-*UAS-5-HT7* (*UAS-5-HT7*) construct. pJFRC28-5XUAS-IVS-GFP-p10 plasmid digested within NotI and XbaI was used to excise the coding sequence of GFP. Using the Gibson Assembly, the complementary DNA (cDNA) of 5-HT7 was cloned in the described plasmid. The right upstream of ATG codon added the Kozak sequence, and the *UAS-5-HT7* construct was injected into the attP40 site using phiC31 integrase-mediated transgenesis. The construct was confirmed using DNA sequencing and PCR. The primers used for cloning *5-HT7* cDNA were as follows:

- UAS-5-HT7-forward:

TCTTATCCTTTACTTCAGGCGGCCGCCACCATGGCTTTATCTGGACAGGACTG

- UAS-5-HT7-reverse:

GTTATTTTAAAAACGATTCATTCTAGATTAAGAGAAAGCTCTCCCTCGC

### Confirmation of Transgenic Flies

**Fig. 1:**
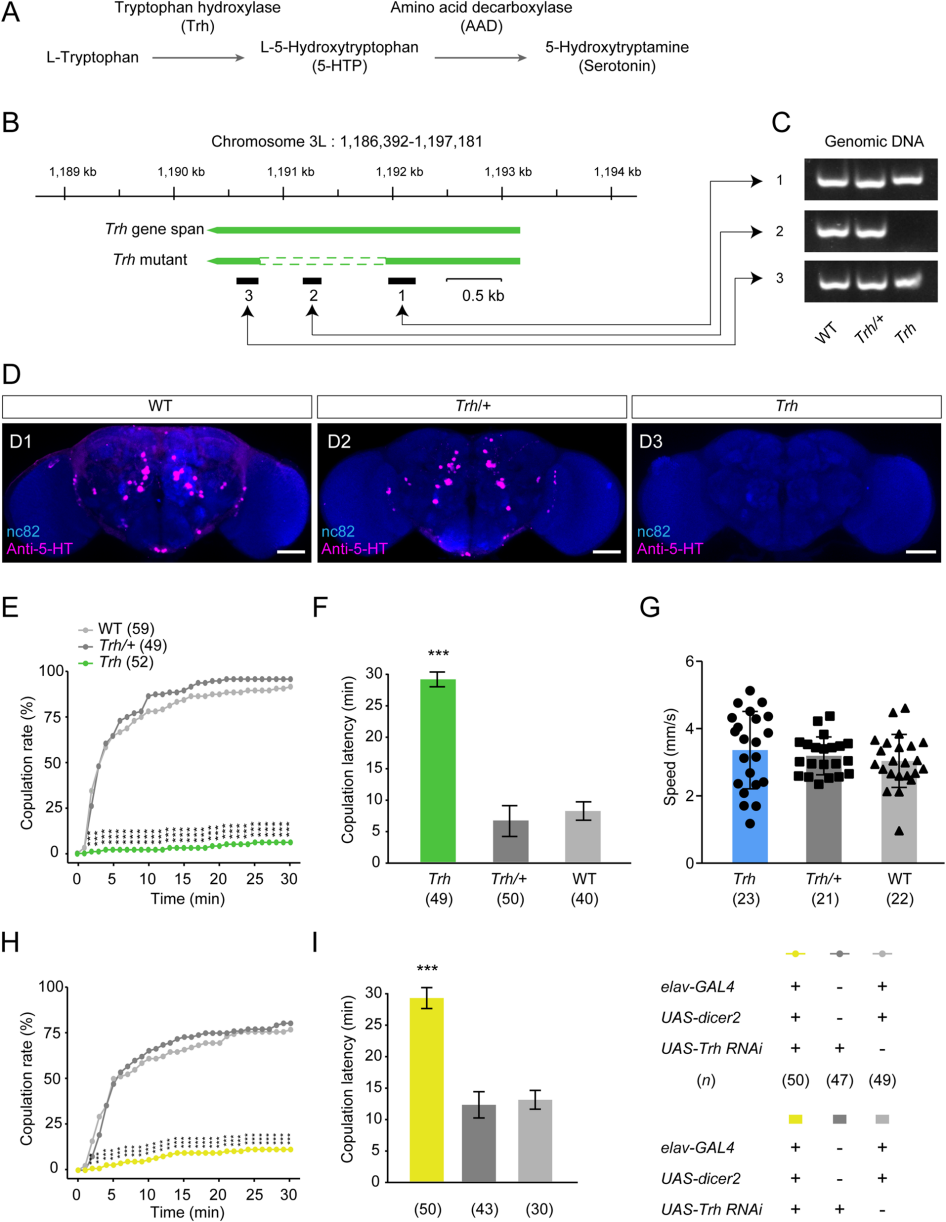
The *Drosophila Trh* gene is essential for virgin female receptivity. **A** 5-HT biosynthesis process. **B** Schemata of the *Trh* gene locus and mutants generated by *Trh* knockout*.* Black bars indicate target regions 1–3 in the PCR analysis in **C**. **C** PCR verification of regions 1–3 in **B** from *Trh* mutant genomic DNA samples. **D** Neuropil expression of the *Trh* gene in female adult brains of wild-type (WT) (**D1**), heterozygous (**D2**), and homozygous *Trh* mutant flies (**D3**), immunostained with anti-5-HT antibody (magenta) and nc82 antibody (blue) (scale bars, 50 μm). **E**, **F** The copulation rate is decreased (**E**) and the copulation latency is prolonged (**F**) in *Trh*-knockout mutants within a 30 min observation period. **G** *Trh* mutants do not differ in locomotor speed from WT and heterozygous control females. **H**, **I** Knockdown of *Trh* expression reduces virgin female receptivity. The copulation rate is decreased (**H**) and the copulation latency is prolonged (**I**) by knocking down *Trh* expression pan-neuronally. ***P <0.001, otherwise no significant difference (*χ*^2^ test for copulation rate; Kruskal-Wallis with *post hoc* Mann-Whitney *U* test for copulation latency). *n* values are shown in parentheses. Error bars, ±SEM.

Genomic DNA was extracted from the whole body of adult flies. Individual flies were crushed with a pestle in 50 µL DNA extraction reagent. After incubation at 95°C for 10 min, the samples were centrifuged at 12,000 r/min for 10 min at room temperature. The supernatant of DNA was collected and added to the mixing system to conduct PCR, according to the manufacturer’s instructions. The mix contained 25 µL 2× MightyAmp buffer, 1 µL MightyAmp DNA polymerase, and 5 µL 10× Additive for high specificity, and was adjusted to 50 µL. Primers used in regions 1–3 (Fig. 1B) were as follows:

- Region 1 forward: GGCTACGGTGGATATTCCAAG
- Region 1 reverse: CATTCAGGCTGTTGTGGAGC
- Region 2 forward: GAGAGGTGGCCTCTGTGAAC
- Region 2 reverse: CGGTGCCCCTTTGAACG
- Region 3 forward: AGGGAACAGATTCTCGGGAC
- Region 3 reverse: ACTTCTTGGTGCAGTGCCTC

### Immunostaining

Flies were dissected in phosphate-buffered saline (PBS), and then the brains were fixed in 2% (weight/volume) paraformaldehyde (PFA) (Electron Microscopy Sciences, Hangzhou, China) for 55 min at room temperature. Then, the samples were washed five times in PBS with 0.3% Triton (PBST) for 15 min and incubated in blocking solution [5% (volume/volume) goat serum (Sigma-Aldrich) diluted in 0.3% PBST] for 1 h at room temperature. The brains were then incubated with the primary antibody (diluted in blocking solution) for >24 h at 4°C, and washed five times in 0.3% PBST for 15 min before incubation with the secondary antibody (1:500, diluted in blocking solution) overnight at 4°C. The samples were washed five times in 0.3% PBST for 15 min and fixed in 4% PFA for >4 h at room temperature. The brains were washed five times with 0.3% PBST for 15 min at room temperature and were placed on a poly-L-lysine-coated coverslip in 0.3% PBST. The brains were then immersed in 30%, 50%, 75%, 95%, and 100% ethanol. The brains were immersed three times in xylene for 5 min and mounted on glass slides using dibutylphthalate polystyrene xylene (DPX) (Sigma-Aldrich) for imaging. Images were generated on a Zeiss 710 confocal microscope (Carl Zeiss, Oberkochen, Germany) and were processed using Fiji software (https://imagej.net/Fiji).

The antibodies used were mouse anti-nc82 (1:50; Developmental Studies Hybridoma Bank, Iowa City, USA), chicken anti-GFP (1:1000; Life technologies, Carlsbad, USA), rabbit anti-RFP (1:500; Invitrogen, Waltham, USA), and rabbit anti-5-HT (1:500; Life technologies). The secondary antibodies were Alexa Fluor goat anti-chicken 488 (1:500; Life technologies), Alexa Fluor goat anti-rabbit 488 (1:500; Life technologies), Alexa Fluor goat anti-mouse 546 (1:500; Life technologies), and Alexa Fluor goat anti-rabbit 633 (1:500; Invitrogen).

### Drug Treatment in Rescue Experiments

**Fig. 2:**
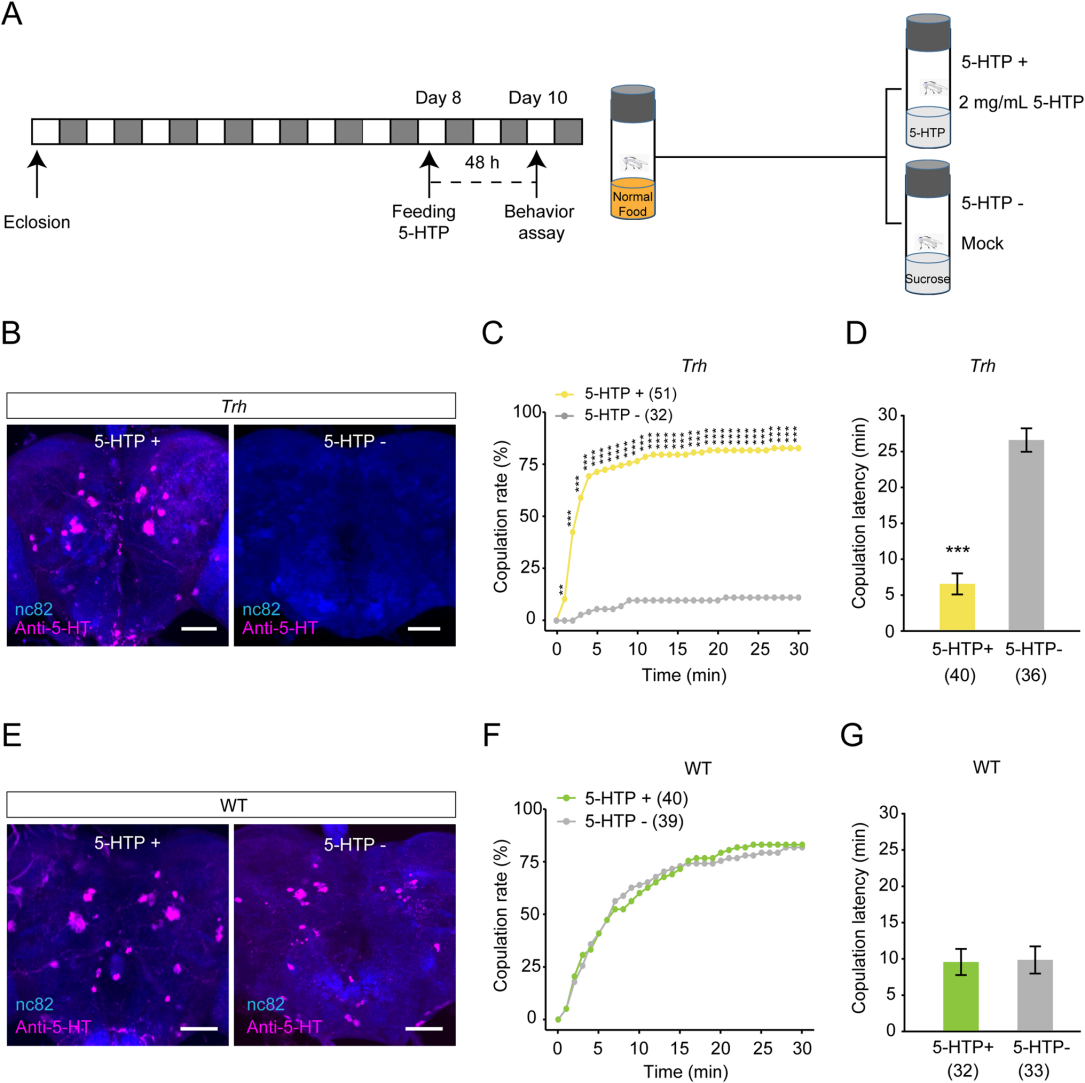
Acutely feeding 5-HTP restores 5-HT expression and virgin female receptivity in *Trh*-knockout mutants. **A** The protocol of the feeding assay in the rescue experiment. **B** Immunoreactivity of 5-HT is restored in the brain of *Trh* mutant females after feeding 5-HTP for 48 h (scale bars, 50 μm). **C**, **D** Virgin female receptivity is restored by feeding *Trh* mutants with 5-HTP. In *Trh*-knockout mutants, the copulation rate is elevated to the normal level (**C**) and the copulation latency is shortened (**D**). **E** Immunoreactivity of 5-HT in wild-type females is not significantly affected by feeding 5-HTP (scale bars, 50 μm). **F**, **G** Sexual receptivity of wild-type virgin females is not affected by 5-HTP feeding. Wild-type females show a comparable copulation rate (**F**) and copulation latency (**G**) with or without 5-HTP feeding. ***P <0.001, **P <0.01, otherwise no significant difference (*χ*^2^ test for copulation rate; Mann-Whitney *U* test for copulation latency). *n* values are shown in parentheses; error bars, ±SEM.

The procedure in the 5-hydroxytryptophan (5-HTP; Sigma-Aldrich) feeding experiment is shown in Fig. 2A. Virgin females were reared on normal food for 8 days after eclosion. Two days before behavioral tests and immunostaining analysis, the flies were divided into two treatment groups. In the control group (5-HTP^−^), females were put on control mock food containing 2% agar and 10% sucrose; in the 5-HTP feeding group (5-HTP^+^), the flies were reared on drug-containing food in which 2 mg/mL 5-HTP was dissolved in the mock food.

### Transcriptional Reporter of Intracellular Ca^2+^ (TRIC) Assay

Virgin females with the genotype *10XUAS-mCD8*::*RFP/13XLexAop2-mCD8*::*GFP*;*nSyb-MKII*::*nlsLexADBDo*;*UAS-p65AD*::*CaM/Trh-GAL4* were collected within 8 h after eclosion until TRIC assay. For mated females, 8-days-old virgin females of given genotypes were transferred to courtship chambers and paired with wild-type male flies. The females that copulated successfully within 30 min were collected for further TRIC analysis. Adult female brains from these two groups (virgin and mated) were dissected, and the whole brain was perfused with a saline solution containing (in mmol/L) 103 NaCl, 3 KCl, 4 MgCl_2_, 1.5 CaCl_2_, 26 NaHCO_3_, 1 NaH_2_PO_4_, 5 N-tri-(hydroxymethyl)-methyl-2-aminoethane-sulfonic acid, 20 glucose, 17 sucrose, and 5 trehalose, adjusted to pH 7.3.

Images were acquired using a confocal microscope (Nikon A1R+, Nikon, Toyko, Japan) with a 40× water immersion objective. The Ca^2+^ signal was indicated by the fluorescence intensity. 488-nm and 546-nm light was used to excite GFP and RFP, respectively. The regions of interest (ROIs) were manually defined in the PLP cluster area and were analyzed using NIS-Elements D (Nikon; https://www.microscope.healthcare.nikon.com/en_EU/products/software). The relative TRIC signal of selected ROIs (GFP signal/RFP signal) was used to compare neural activity in virgin and mated females.

### Statistical Analysis

Statistical analysis and graphics were applied with the R system 4.0.2 (https://www.r-project.org/), MatLab software (MathWorks Inc., MA, USA), and GraphPad Prism 7 software (GraphPad Software, San Diego, USA). The *χ*^2^ test was used to compare the copulation rate of different groups at various time points. The Mann-Whitney *U* test was applied for two-group comparisons. Kruskal-Wallis with the *post hoc* Mann-Whitney *U* test was used to compare the differences between multiple groups. All data are shown as the mean ± SEM. Sample sizes are indicated in the figures. Statistical significance was set at *P* <0.01.

## Results

### 5-HT Modulates Virgin Female Receptivity

In *Drosophila*, it has been found that virgin female receptivity is associated with the release of dopamine (DA), drosulfakinin (DSK), and SIFamide (SIFa) [51–54]. To identify the role of other neurochemicals involved in regulating virgin female receptivity, we screened 108 chemoconnectome (CCT) knockout lines generated by the CRISPR-Cas9 system [49].

Preliminary screening (unpublished data) showed that virgin female receptivity might be regulated by the *Trh* gene that encodes an enzyme catalyzing the first and rate-limiting step of 5-HT biosynthesis (Fig. 1A) [55, 56]. We confirmed the *Trh* knockout line by using PCR analysis at the *Trh* locus in genomic DNA samples (Fig. 1B, C) and by detecting the immunoreactivity of 5-HT in the central brain (Fig. 1D). 5-HT immunoreactivity was found in the brain of wild-type and heterozygous flies (Figs. 1D), but was absent in homozygous *Trh*-knockout flies (Fig. 1D). We found that knockout of *Trh* reduced the virgin female copulation rate and prolonged the copulation latency compared to heterozygous and wild-type control females (Fig. 1E, F). In contrast, we found that *Trh*-knockout females showed locomotor activity comparable to control females (Fig. 1G). Furthermore, knocking down *Trh* expression pan-neuronally using the *elav-GAL4* driver reduced the copulation rate and increased the copulation latency in virgin females (Fig. 1H, I). Given that *Trh* heterozygous and wild-type females displayed similar phenotypes, we mainly used wild-type females as control flies in later experiments.

To determine whether restoration of the 5-HT expression level could rescue the sexual receptivity of *Trh*-knockout females, we performed pharmacological rescue experiments by feeding them with 5-HTP (Fig. 2A). After feeding with 2 mg/mL 5-HTP, 5-HT immunofluorescence was restored in the brain of *Trh* mutant females (Fig. 2B). Furthermore, both copulation rate and copulation latency were rescued to normal levels in *Trh* mutant females (Fig. 2C, D). In contrast, 5-HT immunofluorescence and female sexual receptivity were not significantly affected in wild-type females by feeding with 5-HTP (Fig. 2E–G). These results indicate that 5-HT is crucial for virgin female receptivity in *Drosophila*.

To determine whether 5-HT specifically regulates virgin female receptivity, or affects sexual behavior in both virgin and mated females, we next tested egg-laying and re-mating behaviors in *Trh*-knockout or -knockdown females. We found that *Trh-*knockout virgin females did not show increased egg-laying (Fig. S1A), or any re-mating behavior after mating, like wild-type controls (Fig. S1B). We also observed similar phenotypes in *Trh* RNAi-knockdown females (Fig. S1C, D). Thus, 5-HT specifically regulates virgin female receptivity but not post-mating behaviors.

### ***Trh***^***+***^ Neurons Regulate Virgin Female Receptivity

**Fig. 3:**
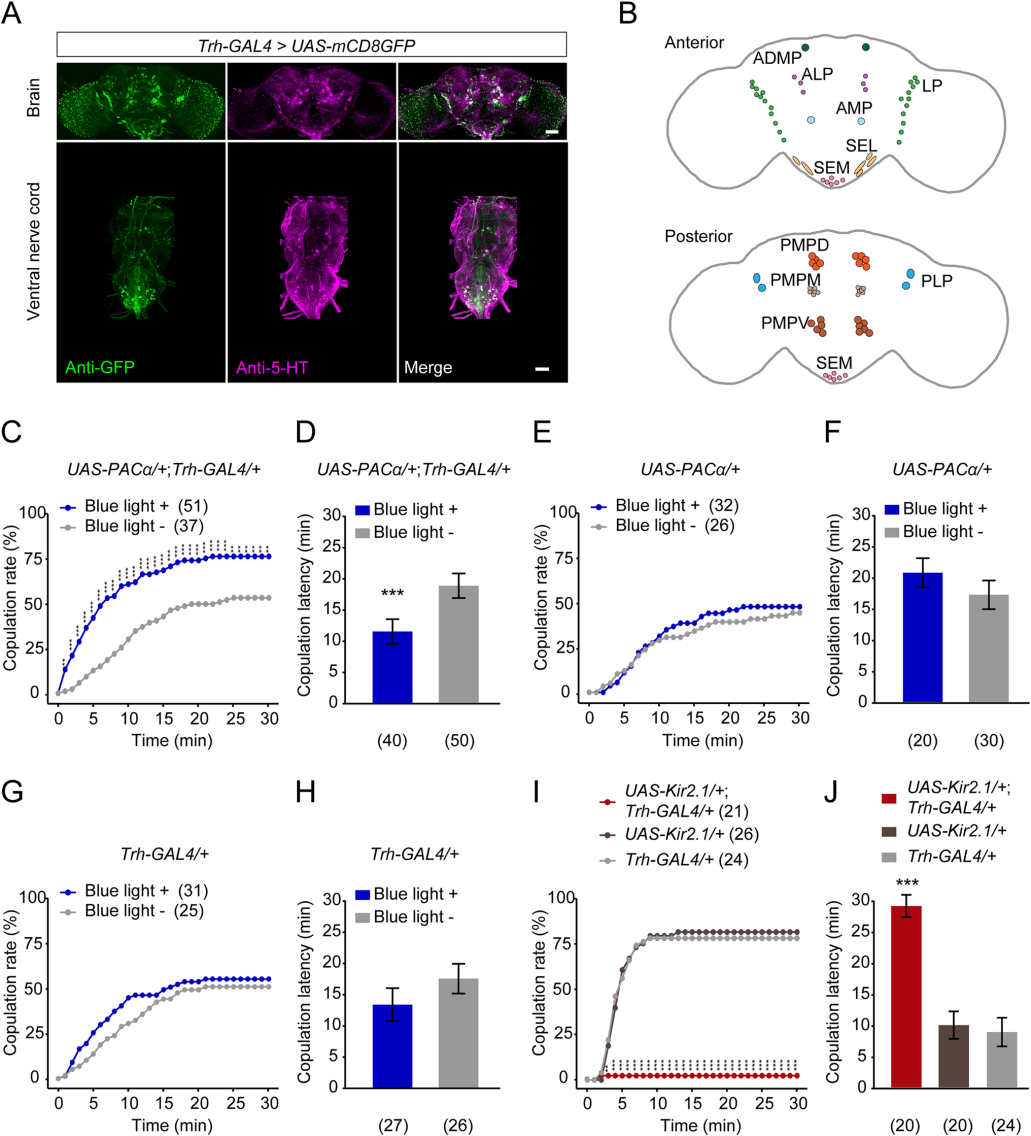
*Trh*^*+*^ neurons regulate virgin female receptivity. **A** Expression pattern of the *Trh* gene and *Trh-GAL4* visualized with anti-5-HT antibody (magenta) and anti-GFP (green) in a *UAS-mCD8-GFP/Trh-GAL4* female brain and VNC (scale bars, 50 μm). **B** Anterior (upper) and posterior views (lower) of *Trh*^*+*^ neurons labeled by *Trh-GAL4* in the brain. **C**, **D** The copulation rate is increased (**C**) and the copulation latency is shortened (**D**) after blue light stimulation in *UAS-PACα/Trh-GAL4* virgin females. **E**, **F** *UAS-PACα/+* control females do not display a significantly changed copulation rate (**E**) and copulation latency (**F**) after blue light stimulation. **G**, **H** *Trh-GAL4/+* control females show a comparable copulation rate (**G**) and copulation latency (**H**) with or without blue light stimulation. **I**, **J** *UAS-Kir2.1/Trh-GAL4* virgin females exhibit a decreased copulation rate (**I**) and prolonged copulation latency (**J**) relative to control females. ***P <0.001, **P <0.01, otherwise no significant difference (*χ*^2^ test for copulation rate analysis; Mann-Whitney *U* test for **D**, **F,** and **H**; Kruskal-Wallis with *post hoc* Mann-Whitney *U* test for **J**). *n* values are shown in parentheses; error bars, ±SEM. ADMP, anterior dorsomedial protocerebrum; ALP, anterior lateral protocerebrum; AMP, anterior medial protocerebrum; LP, lateral protocerebrum; PLP, posterior lateral protocerebrum; PMPD, dorsal, PMPM, medial, and PMPV, ventral posterior medial protocerebrum; SEL, lateral and SEM, medial subesophageal ganglion.

We used a *UAS-mCD8GFP* reporter to visualize the expression pattern of the newly-generated *Trh-GAL4* [36] (Fig. 3A). The *Trh-GAL4* labeled 5-HT clusters in the brain similar to those reported by previous studies [57, 58]. We mapped the distribution of *Trh*^*+*^ neurons in the central brain including the anterior dorsomedial protocerebrum (ADMP), anterior lateral protocerebrum (ALP), anterior medial protocerebrum (AMP), lateral subesophageal ganglion (SEL), lateral protocerebrum (LP), medial subesophageal ganglion (SEM), posterior medial protocerebrum, dorsal (PMPD), medial (PMPM), and ventral (PMPV) posterior medial protocerebrum, and posterior lateral protocerebrum (PLP) (Fig. 3B).

We then analyzed whether *Trh*^*+*^ neurons are involved in the modulation of female sexual receptivity. We activated all *Trh*-expressing neurons by using PACα [59], which specifically enhances intracellular cAMP levels after blue light stimulation, and also has a more moderate activation effect than *CsChrimson* [60] or *dTrpA1* [61], since the *Trh*-GAL4 labels a large number of neurons and strong activation of these neurons may have side-effects. We found that activation of *Trh-GAL4* neurons increased copulation rate and decreased copulation latency in *UAS-PACα/Trh-GAL4* virgin females (Fig. 3C, D). In contrast, there was no significant change in copulation rate and copulation latency after blue light stimulation in *UAS-PACα/+* or *Trh-GAL4/+* control females (Fig. 3E–H).

To further confirm whether *Trh*^*+*^ neurons are necessary for virgin female receptivity, we silenced these neurons by expressing the inwardly-rectifying K^+^ channel (Kir2.1) [62]. *UAS-Kir2.1/Trh-GAL4* virgin females exhibited a dramatic reduction in copulation rate and a prolonged copulation latency compared to control females (Fig. 3I, J). We also found that blocking neurotransmission from *Trh*^*+*^ neurons expressing the temperature-sensitive *shibire*^*ts*^ (*shi*^*ts*^) [63] significantly reduced virgin female receptivity (Fig. S2). Furthermore, neither activation nor inactivation of *Trh*^*+*^ neurons affected sexual receptivity in mated females (Tables S1, S2). Taken together, our findings indicate that the activity of *Trh*^*+*^ neurons positively regulates sexual receptivity in virgin, but not mated females.

### ***Trh***^***+***^***fru***^***+***^neurons Mediate Virgin Female Receptivity

**Fig. 4:**
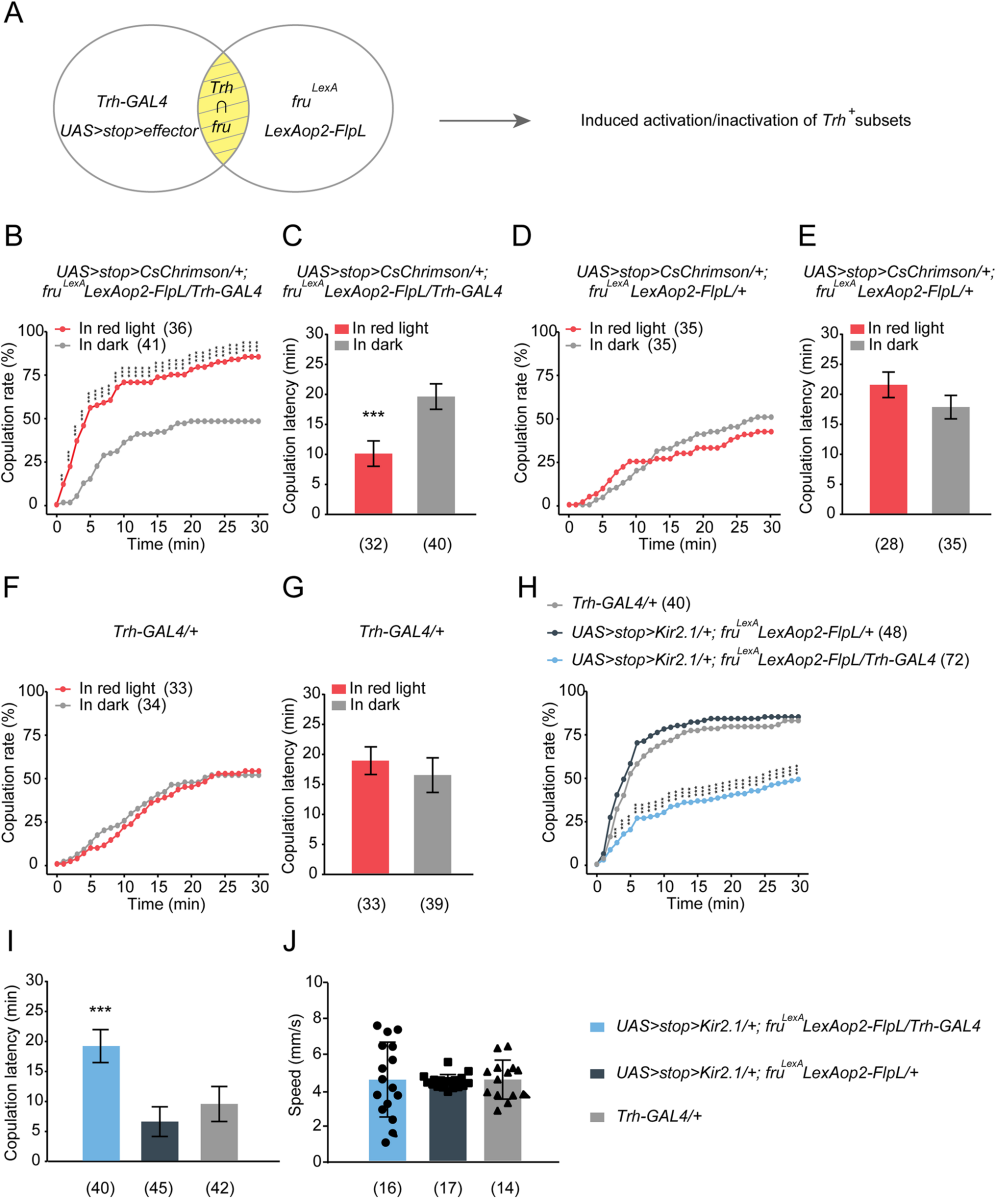
*Trh*^*+*^*fru*^*+*^ neurons mediate virgin female receptivity. **A** The intersectional strategy used to narrow down the serotonergic neurons. FLP is driven by *fru*^*LexA*^*LexAop* to excise the transcriptional stop cassette, allowing the expression of specific effectors in intersecting neurons (*Trh*∩*fru*). **B**, **C** Activation of *Trh*^*+*^*fru*^*+*^ neurons with red light increases the copulation rate (**B**) and decreases the copulation latency (**C**) in *UAS>stop>CsChrimson*; *fru*^*LexA*^* LexAop2-FlpL/Trh-GAL4* virgin females. **D–G** *UAS>stop>CsChrimson*; *fru*^*LexA*^* LexAop2-FlpL/+* or *Trh-GAL4/+* control virgin females do not show a red light-induced change in copulation rate or copulation latency. **H**, **I** Silencing the *Trh*^*+*^*fru*^*+*^ neurons decreases copulation rate (**H**) and prolongs copulation latency (**I**) in virgin females compared with control females. **J** Inactivation of *Trh*^*+*^*fru*^*+*^ neurons in these females does not affect locomotor speed. ***P <0.001, **P <0.01, otherwise no significant difference (*χ*^2^ test for copulation rate; Mann-Whitney *U* test for **C**, **E,** and **G**; Kruskal-Wallis with *post hoc* Mann-Whitney *U* test for **I** and **J**). *n* values shown in parentheses; error bars, ±SEM.

We next set out to narrow down the serotonergic neurons that promote virgin female receptivity. As previous studies revealed crucial roles of *fru* or *dsx* neurons in regulating female receptivity [15, 41, 46], we tried to specifically label and manipulate *Trh*^*+*^∩*fru*^*+*^ or *Trh*^*+*^∩*dsx*^*+*^ neurons. We first applied the FLP/FRT intersectional strategy [64] to restrict expression in overlapping *Trh*^*+*^ and *fru*^*+*^ neurons (Fig. 4A). *UAS>stop>CsChrimson*; *fru*^*LexA*^* LexAop2-FlpL/Trh-GAL4* virgin females, in which the overlapping *Trh*^*+*^ and *fru*^*+*^ neurons (referred to as *Trh*^*+*^*fru*^*+*^ neurons hereafter) express the optogenetic effector *CsChrimson* [60], displayed a much higher copulation rate and decreased copulation latency with red light stimulation (Fig. 4B, C). In contrast, control *UAS>stop>CsChrimson*; *fru*^*LexA*^* LexAop2-FlpL/+*, or *Trh-GAL4/+* virgin females did not exhibit red light-induced changes in receptivity (Fig. 4D–G). We also used the thermogenetic effector *TrpA1* [61] to activate the *Trh*^*+*^*fru*^*+*^ neurons, and found that heat-induced activation of *Trh*^*+*^*fru*^*+*^ neurons slightly but significantly enhanced virgin female receptivity (Fig. S3). These results indicate that activation of *Trh*^*+*^*fru*^*+*^ neurons is able to promote virgin female receptivity.

We next tried to silence the *Trh*^*+*^*fru*^*+*^ neurons using the same intersectional strategy to express the inwardly-rectifying K^+^ channel Kir2.1 [62]. Silencing the *Trh*^*+*^*fru*^*+*^ neurons dramatically reduced copulation rate and increased copulation latency in virgin females compared with control females (Fig. 4H, I). The reduction of receptivity in these females was not due to locomotor activity as they displayed a walking speed comparable with control females (Fig. 4J). We also used the same intersectional strategy to silence the overlapping *Trh*^*+*^ and *dsx*^*+*^ neurons, but did not find any significant change in virgin female receptivity (Fig. S4A, B). Thus, these findings indicate that *Trh*^*+*^*fru*^*+*^ neurons are crucial for virgin female receptivity.

### Sexually Dimorphic ***Trh***^***+***^***fru***^***+***^ PLP Neurons Promote Virgin Female Receptivity

**Fig. 5:**
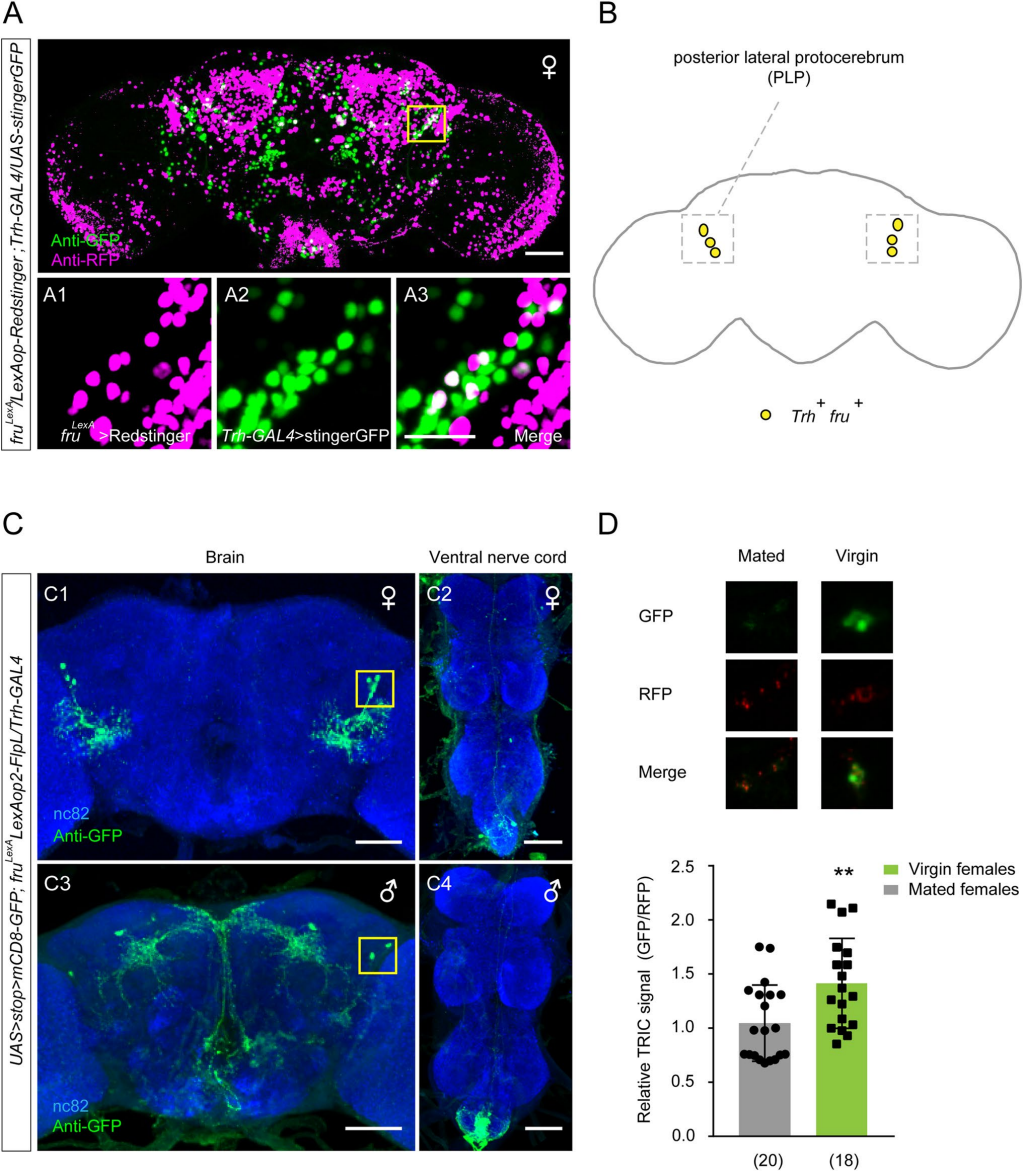
Identification of sexually dimorphic *Trh*^*+*^*fru*^*+*^ PLP neurons. **A** The cell cluster of PLP neurons (yellow box) is labeled by *Trh-GAL4/UAS-stingerGFP* (green) and *fru*^*LexA*^/*LexAop-Redstinger* (magenta) [scale bars, 50 μm (upper) and 20 μm (lower)]. **B** Schematic of overlapped *Trh*^*+*^*fru*^*+*^ PLP neurons (yellow dots) in the brain of females. **C** Neurons co-expressing *Trh*^*+*^ and *fru*^*+*^ visualized with anti-GFP (green) in females and males. Anti-nc82 (blue) indicates the neuropil of the central nervous system. Yellow boxes indicate cell bodies of the *Trh*^*+*^*fru*^*+*^ PLP neurons (scale bars, 50 μm). **D** The TRIC signal in PLP neurons is significantly stronger in virgin females than that in mated females. **P <0.01 (Mann-Whitney *U* test). *n* values shown in parentheses; error bars, ±SEM.

The above results demonstrated a crucial role of *Trh*^*+*^*fru*^*+*^ neurons in regulating virgin female receptivity. To visualize the *Trh*^*+*^*fru*^*+*^ neurons, we first applied double-labeling in *Trh-GAL4/UAS-stingerGFP*; *fru*^*LexA*^*/LexAop-Redstinger* flies, and found that a subset of PLP neurons was co-labeled by *Trh*^*+*^ and *fru*^*+*^ in the brain of females (Fig. 5A, B). In addition, we applied the FLP/FRT intersectional strategy to express GFP in both sexes of *UAS>stop>mCD8-GFP*; *fru*^*LexA*^* LexAop2-FlpL/Trh-GAL4* flies. We observed GFP expression in ~3 pairs of PLP neurons (Fig. 5C) and a few neurons in the ventral nerve cord (VNC) in females (Fig. 5C); in contrast, we observed 1–2 pairs of PLP neurons as well as a few other neurons in the brain (Fig. 5C) and VNC in males (Fig. 5C). Thus, there might be female-specific *Trh*^*+*^*fru*^*+*^ PLP neurons that regulate virgin female receptivity. We also used the same strategy to visualize *Trh*^+^*dsx*^*+*^ neurons, and observed sparse expression in the brain and VNC (Fig. S4C). Nevertheless, these *Trh*^+^*dsx*^*+*^ neurons were not involved in virgin female receptivity (Fig. S4A, B). In addition, we used the above strategy to express nsyb-GFP and Dscam-GFP and localized presynaptic and postsynaptic sites of the *Trh*^*+*^*fru*^*+*^ PLP neurons in females (Fig. S5).

To further test the role of *Trh*^*+*^*fru*^*+*^ PLP neurons in female receptivity, we monitored the neural activity of PLP neurons by expressing TRIC [65] in virgin and mated females. The TRIC signal in PLP neurons was significantly stronger in virgins than that in mated females (Fig. 5D). Thus, the spontaneous activity of *Trh*^*+*^*fru*^*+*^ PLP neurons is higher in virgin females, which might reflect an internal state of female receptivity. Together, these results demonstrate that a subset of sexually dimorphic *Trh*^*+*^*fru*^*+*^ PLP neurons promotes sexual receptivity in virgin females.

### 5-HT_1A_ and 5-HT_7_ Receptors Regulate Virgin Female Receptivity

**Fig. 6:**
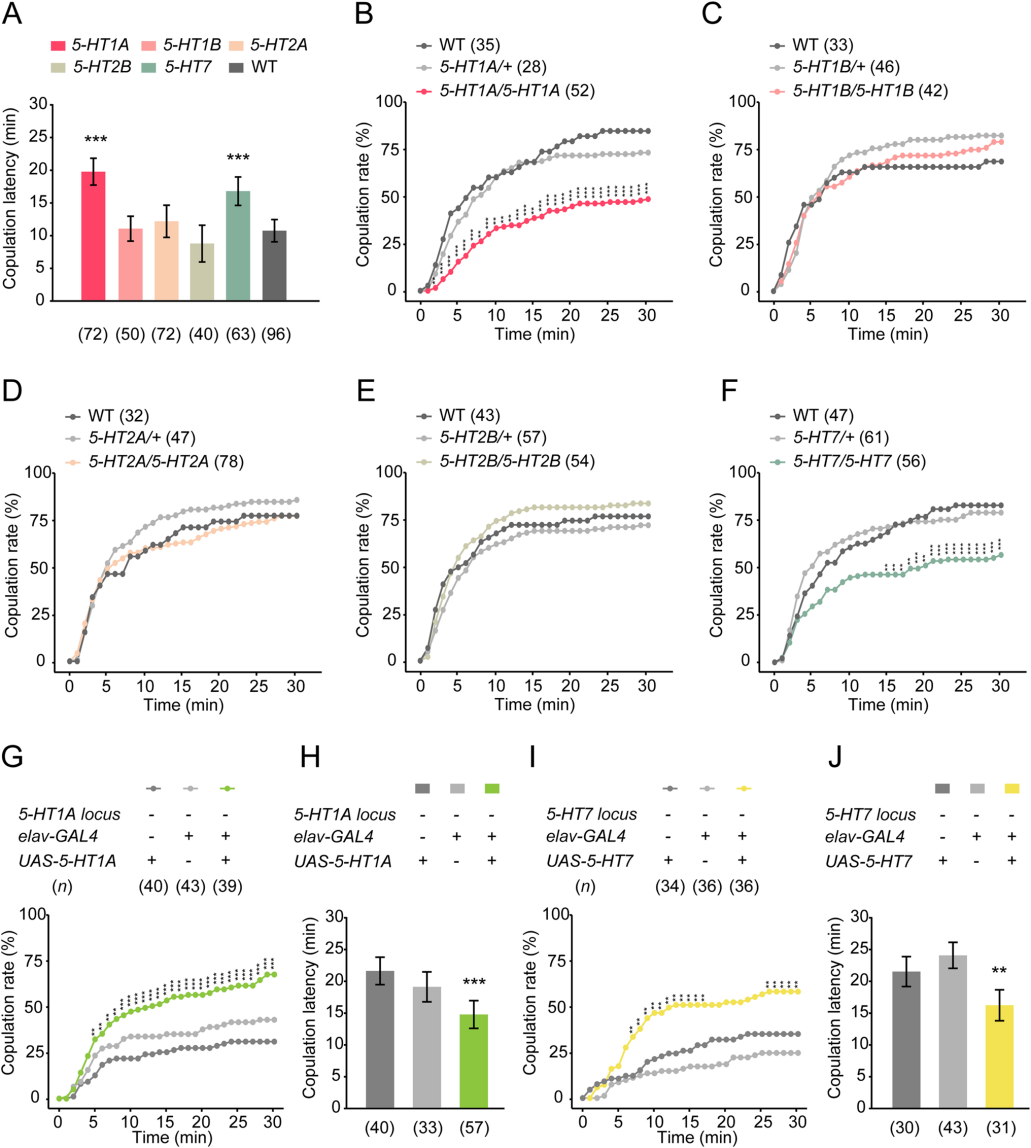
5-HT_1A_ and 5-HT_7_ receptors are involved in virgin female receptivity. **A** The copulation latency is extended in *5-HT1A*- and *5-HT7*-knockout females compared to wild-type control females. **B–F** Copulation rate of females with 5-HT receptor knockout during a 30-min observation period. Knockout of *5-HT1A* (**B**) and *5-HT7* (**F**), but not *5-HT1B* (**C**), *5-HT2A* (**D**), or *5-HT2B* (**E**), significantly reduces the copulation rate in virgin females. **G**, **H** The copulation rate (**G**) is increased, and the copulation latency (**H**) is reduced in *5-HT1A* mutant females with the *elav-GAL4*-driven expression of *5-HT1A*. **I**, **J** The *elav-GAL4*-driven expression of *UAS-5-HT7* in *5-HT7*-knockout females also restores the copulation rate (**I**) and copulation latency (**J**) to normal levels. ***P <0.001, **P <0.01, otherwise no significant difference (Kruskal-Wallis with *post hoc* Mann-Whitney *U* test for copulation latency; *χ*^2^ test for copulation rate). *n* values are shown in parentheses; error bars, ±SEM.

There are five types of 5-HT G-protein-coupled receptors (GPCRs): 5-HT_1A_, 5-HT_1B_, 5-HT_2A_, 5-HT_2B_, and 5-HT_7_ [36, 66–68]. These receptors, which are mammalian orthologs of the 5-HT receptor family, are expressed widely in the central nervous system [69] (Fig. S6), and regulate complex behaviors [37, 70–72]. To identify which 5-HT receptors are involved in virgin female receptivity, we used knockout lines of each 5-HT receptor and found that the copulation latency of *5-HT1A*- and *5-HT7*-knockout females was significantly longer than in the wild-type control females (Fig. 6A). In addition, knockout of *5-HT1A* and *5-HT7*, but not *5-HT1B, 5-HT2A*, or *5-HT2B*, significantly reduced the copulation rate in virgin females (Fig. 6B–F).

Given that female sexual receptivity was reduced in the *5-HT1A* and *5-HT7* mutants, we analyzed whether overexpressing *5-HT1A* or *5-HT7* in *5-HT1A* or *5-HT7* mutants could restore female receptivity. We used *elav-GAL4* to drive the expression of *UAS-5-HT1A* or *UAS-5-HT7* in *5-HT1A* or *5-HT7* mutant flies, respectively. We found that the copulation rate was increased, and copulation latency was reduced in *5-HT1A* mutant females with *elav-GAL4*-driven expression of *5-HT1A* (Fig. 6G, H). Meanwhile, the copulation rate and the copulation latency of *5-HT7* knockout females were also restored to normal levels with *elav-GAL4*-driven expression of *5-HT7* (Fig. 6I, J). These results suggest that 5-HT_1A_ and 5-HT_7_ receptors are involved in virgin female receptivity.

## Discussion

In animals, males often initiate courtship, and females decide whether to accept or reject copulation. Acceptance by females is a prerequisite for reproductive success, which is determined not only by external factors but also by internal sexual motivation. Monoamine neurotransmitters and neuropeptides have been found to regulate female receptivity, such as dopamine [51, 53], octopamine [27], DSK [54], and SIFa [52]. Here, we showed that 5-HT signaling plays a critical role in virgin female receptivity. Both the knockout and *RNAi* knockdown of *Trh* reduced the receptivity. 5-HT may regulate virgin female receptivity through two of the 5-HT receptors, 5-HT_1A_ and 5-HT_7_. Furthermore, we identified ~3 pairs of sexually dimorphic *Trh*^*+*^*fru*^*+*^ PLP neurons in the female brain that promote sexual receptivity in virgin females.

5-HT is a well-known conserved molecule, which participates in regulating sexual behavior in a wide range of species [73]. In mammals, a fraction of 5-HT is produced in the central nervous system to regulate male sexual behavior, such as ejaculation and orgasm [74–77]. Moreover, 5-HT is required for male sexual preference: male mice lacking 5-HT prefer to court males rather than females [78]. Although the role of 5-HT has been unraveled in the modulation of male sexual behavior, little is known about its role in female sexual behavior. We found that *Trh* knockout females showed a dramatic reduction in receptivity, which was rescued by acutely feeding 5-HTP before the receptivity assay. Loss of 5-HT specifically impaired virgin female receptivity but not post-mating behaviors. Furthermore, the spontaneous activity of a subset of 5-HT-releasing neurons was stronger in receptive virgin females. We speculate that 5-HT is required to maintain proper activity in sex-promoting neurons, and thus serves as a positive regulator for sexual motivation in virgin females.

All 5-HT receptors, 5-HT_1A_, 5-HT_1B_, 5-HT_2A_, 5-HT_2B_, and 5-HT_7_, play coordinated roles in serotonin signaling to modulate diverse complex behaviors including aggression, locomotion, and sleep [36, 79, 80]. Notably, knockout of either *Trh* or individual 5-HT receptors did not result in any evident developmental deficit in flies, which suggests that the role of 5-HT signaling in a variety of behaviors is not due to a developmental deficit. We found that the 5-HT_1A_ and 5-HT_7_ receptors, but not the 5-HT_1B_, 5-HT_2A_, or 5-HT_2B_ receptors, are involved in virgin female receptivity. Knockout of 5-HT_1A_ or 5-HT_7_ reduced the receptivity, although not as severely as knockout of *Trh*, suggesting that 5-HT receptors might have parallel and redundant roles in virgin female receptivity. We also noted that knockout of 5-HT_2B_ induced a slight increase in the female copulation rate. It has been reported that 5-HT_2B_ regulates the amount of sleep and sleep homeostasis [36, 81], while sleep significantly influences female mating behaviors. Whether 5-HT_2B_ functions to coordinate female sleep and sexual behavior awaits further investigation.

Approximately 90 serotonergic neurons are present in the central brain and are divided into several clusters into distinct brain regions [57, 58]. Distinct clusters of serotonergic neurons modulate various behaviors, such as walking, long-term memory, and feeding [38, 69]. Previous reports have indicated that female sexual receptivity is regulated by *fru*^*+*^ neurons [23, 46, 82], which encouraged us to subdivide the serotonergic neurons involved in sexual receptivity by intersecting with *fru*^*+*^ neurons. We identified *Trh*^*+*^*fru*^*+*^ neurons in the PLP cluster to be crucial for virgin female receptivity. Interestingly, there were more *Trh*^*+*^*fru*^*+*^ PLP neurons in females than in males, suggesting the involvement of female-specific *Trh*^*+*^*fru*^*+*^ PLP neurons in female receptivity. Such dimorphism of *Trh*^*+*^*fru*^*+*^ PLP neurons might be regulated by the presence/absence of Fru^M^ protein in males and females, respectively, as found in other sexually dimorphic *fru*^*+*^ neurons [83–85]. We also found that 5-HT functions in sexually dimorphic neurons to mediate male courtship behavior (unpublished data). Thus, 5-HT signaling regulates both male and female sexual behavior through sexually dimorphic neural circuits. Future studies may reveal how 5-HT functions in each sex to mediate different aspects of sexual behavior, possibly through distinct 5-HT receptors.

## Supplementary Information

Below is the link to the electronic supplementary material.

Supplementary file1 (PDF 1030 kb)
