# Supplementary material for: Serotonin Signaling Modulates Sexual Receptivity of Virgin Female Drosophila
Source: Neurosci Bull. 2022 Jul 5;38(11):1277–91. doi: 10.1007/s12264-022-00908-8 (PMC9672162; doi:10.1007/s12264-022-00908-8)
Supplement: Supplementary file 1 — Supplementary file1 (PDF 1030 kb) [file 12264_2022_908_MOESM1_ESM.pdf]

Supplementary Materials

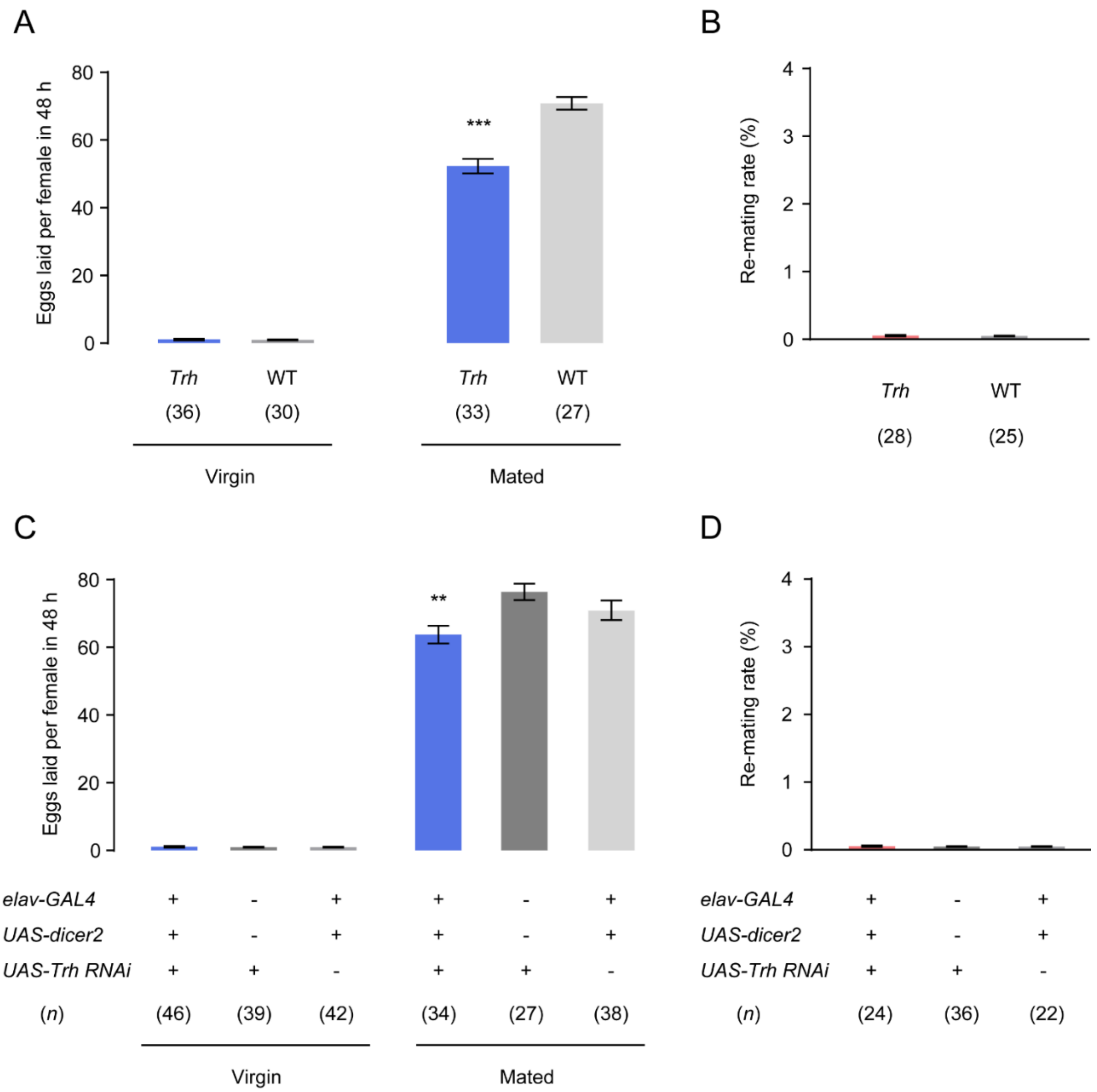

Fig. S1 Knockout or knockdown of *Trh* does not trigger the female post-mating response. **A** Virgin females with *Trh* knockout do not show increased egg-laying during a 48 h observation period. **B** Knockout of *Trh* has no effect on the re-mating rate. **C** Knockdown of *Trh* does not induce increased egg-laying in virgin females. **D** Virgin females with *Trh* knockdown do not show any re-mating behavior after mating. ***P <0.001, **P <0.01, otherwise no significant difference (Mann-Whitney U test for **A** and **B**; Kruskal-Wallis with *post hoc* Mann-Whitney U test for **C** and **D**). n values are shown in parentheses; error bars, ±SEM).

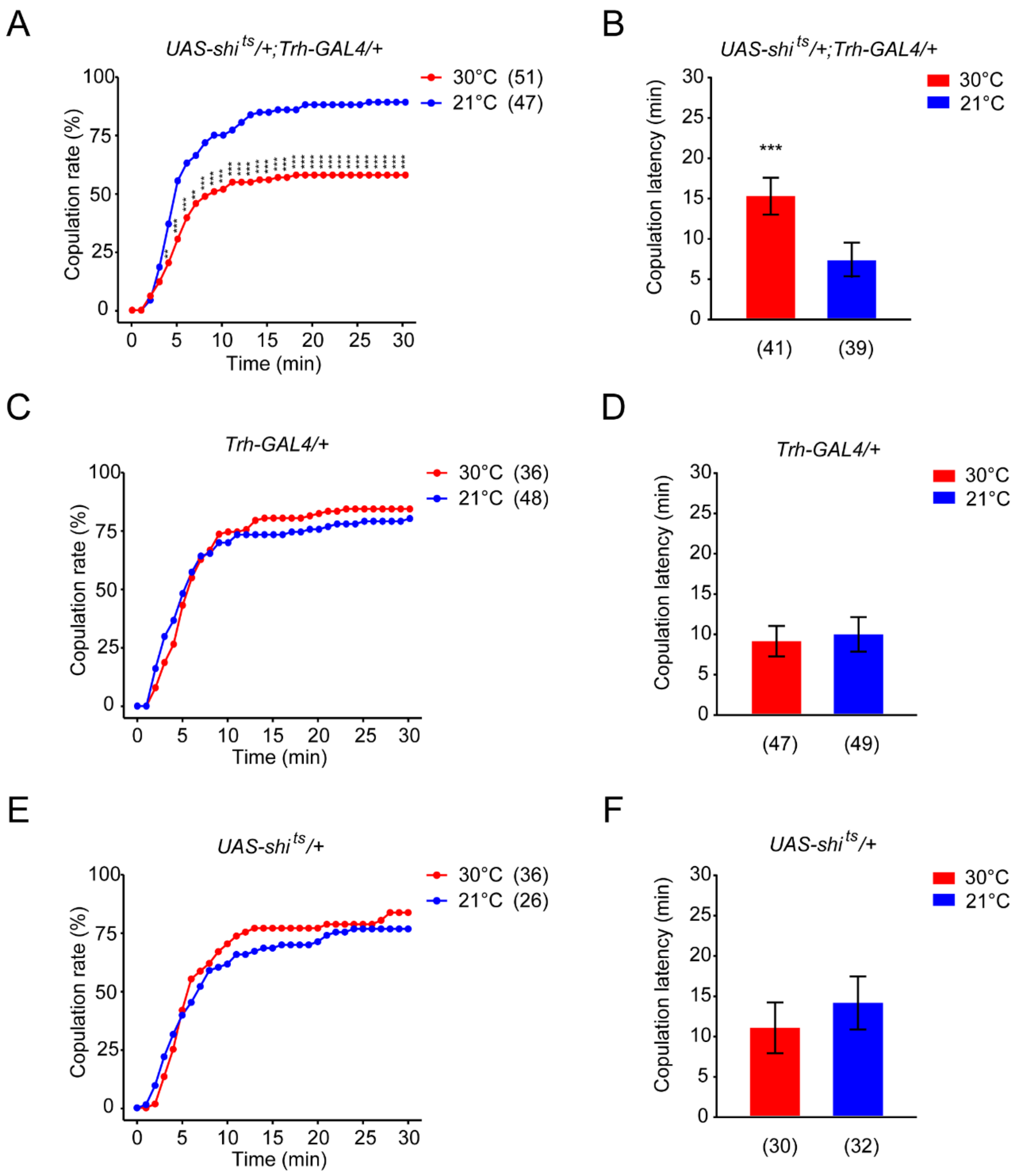

Fig. S2 Silencing *Trh*$^{+}$ neurons reduces sexual receptivity in virgin females. **A, B** *UAS-shi*^{ts}/*Trh-GAL4* virgin females show a reduction in copulation rate (**A**) and an increase in copulation latency (**B**) at 30°C compared with control females at the permissive temperature of 21°C. **C–F** No significant change in the copulation rate and copulation latency occurs in *Trh-GAL4/+* or *UAS-shi*^{ts}/+ control females between 21°C and 30°C. ***P <0.001, **P <0.01, otherwise no significant difference (χ^2 test for **A**, **C,** and **E**; Mann-Whitney U test for **B**, **D,** and **F**). n values are shown in parentheses; error bars, ±SEM.

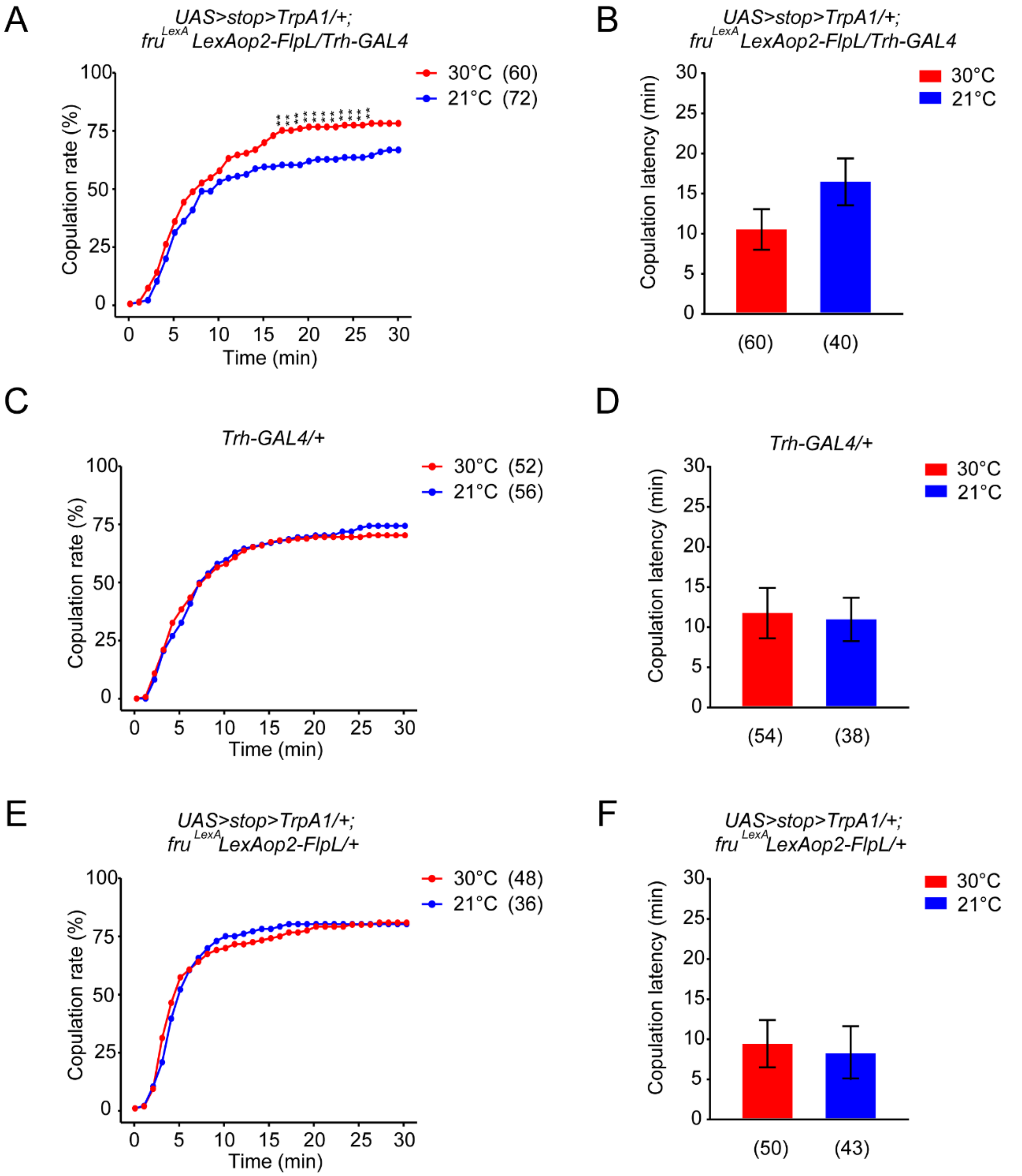

Fig. S3 Thermogenetic activation of *Trh*$^+$*fru*$^+$ neurons enhances virgin female receptivity. **A**, **B** Heat-induced activation of *Trh*$^+$*fru*$^+$ neurons increases female copulation rate (**A**) and slightly shortens copulation latency (**B**). **C–F** Control *Trh-GAL4/+* or *UAS>stop>TrpA1; fru*^{LexA} *LexAop2-FlpL/+* virgin females do not exhibit a temperature-induced change in copulation rate or copulation latency. ***P* <0.01, otherwise no significant difference (χ^2 test for **A**, **C,** and **E**; Mann-Whitney *U* test for **B**, **D,** and **F**). *n* values are shown in parentheses; error bars, ±SEM.

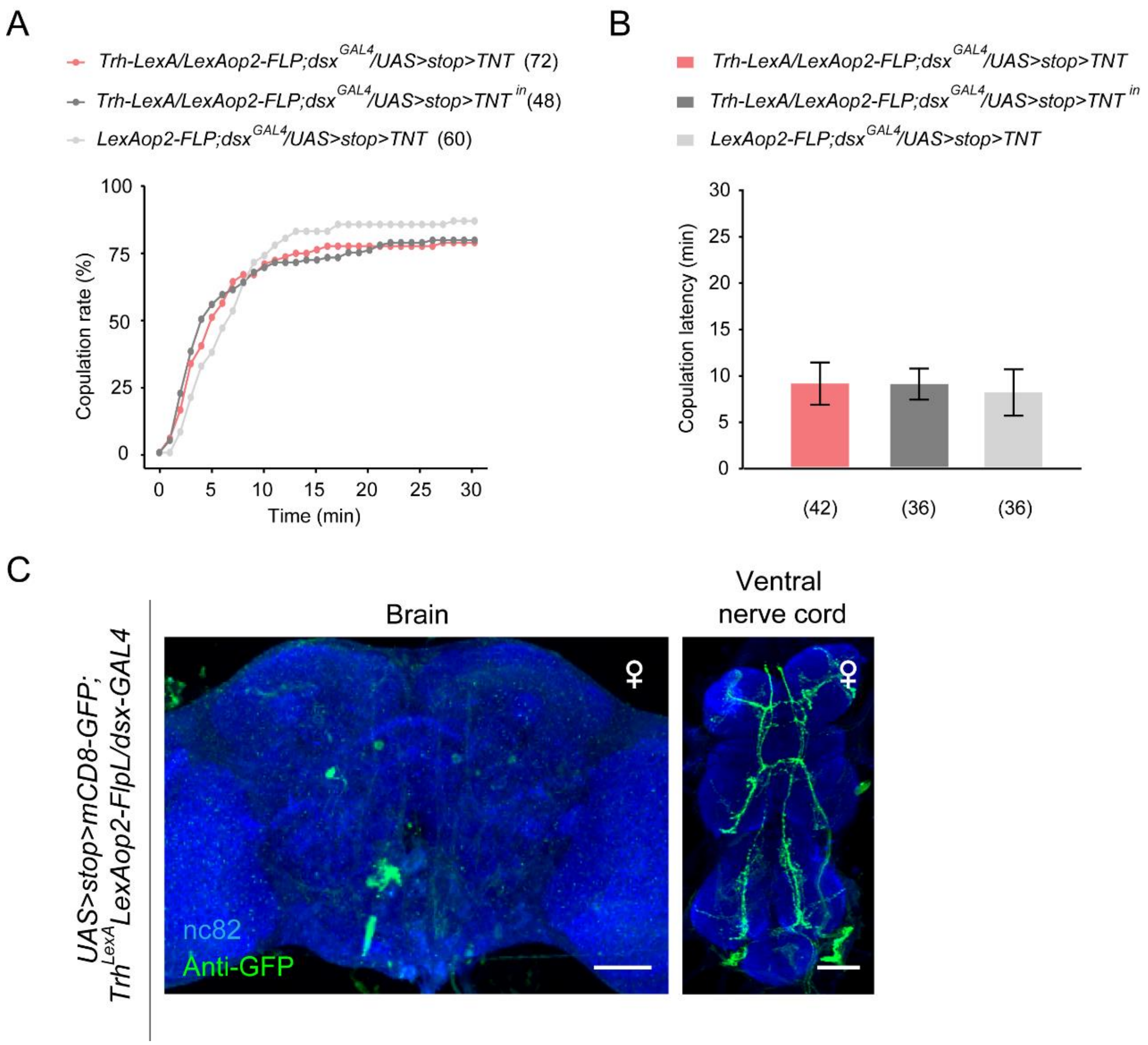

Fig. S4 *Trh*$^{+}$*dsx*$^{+}$ neurons are not involved in virgin female receptivity. **A**, **B** Silencing *Trh*$^{+}$*dsx*$^{+}$ neurons in virgin females does not induce any significant change in copulation rate (**A**) or copulation latency (**B**). **C** Intersection of *Trh*$^{+}$ and *dsx*$^{+}$ neurons expressing GFP stained with anti-GFP (green) and anti-nc82 (blue) in the nervous system (scale bars, 50 μm). χ^2 test for **A**; Kruskal-Wallis with *post hoc* Mann-Whitney *U* test for **B**. *n* values are shown in parentheses; error bars, ±SEM.

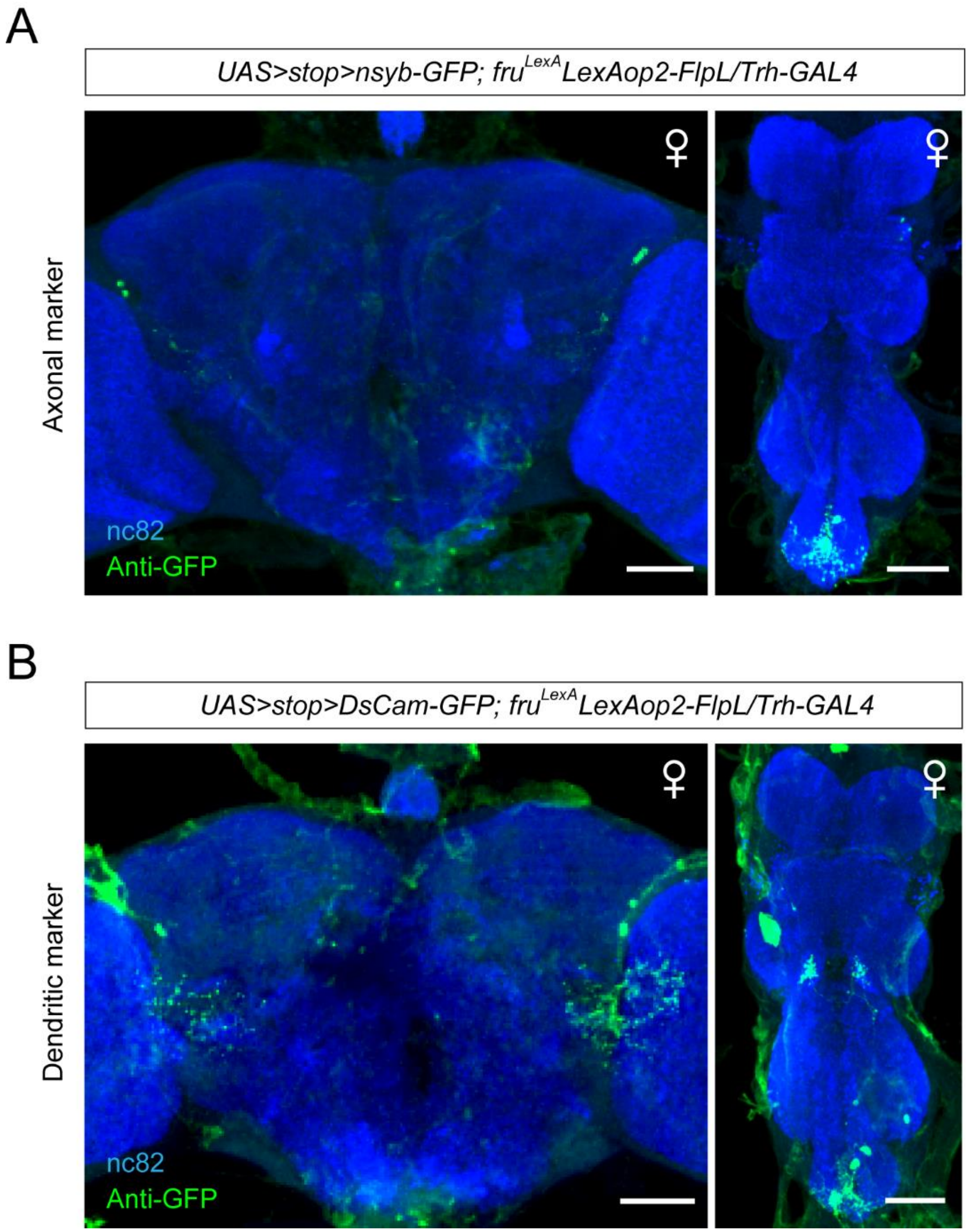

Fig. S5 Axonal and dendritic expression of *Trh*$^{+}$*fru*$^{+}$ PLP neurons. **A**, **B** Intersection of *Trh*$^{+}$ and *fru*$^{+}$ neurons expressing nsyb-GFP and Dscam-GFP in females stained with anti-GFP (green) and anti-nc82 (blue). nsyb-GFP indicates axonal expression (**A**) and Dscam-GFP indicates dendritic expression (**B**) (scale bars, 50 μm).

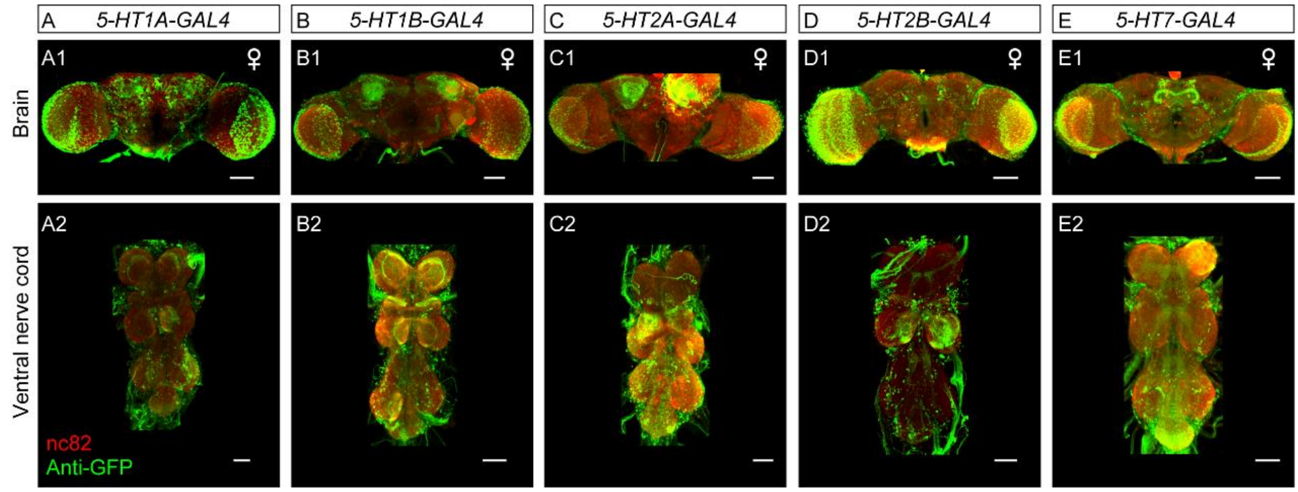

Fig. S6 Expression patterns of 5-HT receptors: 5-HT$_{1A}$, 5-HT$_{1B}$, 5-HT$_{2A}$, 5-HT$_{2B}$, and 5-HT$_{7}$. **A–E** Expression of 5-HT receptors visualized with anti-GFP (green) by knock-in GAL4s expressing *UAS-mCD8GFP* in the female brain (**A1–E1**) and VNC (**A2–E2**) (scale bars, 50 μm).

Table S1 Activation of *Trh*$^{+}$ neurons does not affect sexual receptivity in mated females

	UAS-PACα/+;Trh-GAL4/+		*UAS-PACα/+*		*Trh-GAL4/+*	
Blue light	+	−	+	−	+	−
Re-mating pairs	0/52	0/36	0/26	0/16	0/37	0/33

The numbers in the table indicated the number of re-mating pairs during a 1-h observation period divided by the total number of tested pairs.

Table S2 Inactivation of *Trh*$^{+}$ neurons does not affect sexual receptivity in mated females

	UAS-shi^{ts}/+;Trh-GAL4/+		*UAS-shi^{ts}/+*		*Trh-GAL4/+*	
Temperature	30°C	21°C	30°C	21°C	30°C	21°C
Re-mating pairs	0/42	1/26	0/25	0/23	0/31	0/15

The numbers in the table indicate the number of re-mating pairs during a 1-h observation period divided by the total number of tested pairs.
